# Functional mapping of the fission yeast DNA polymerase δ B-subunit Cdc1 by site-directed and random pentapeptide insertion mutagenesis

**DOI:** 10.1186/1471-2199-10-82

**Published:** 2009-08-17

**Authors:** Javier Sanchez Garcia, Andrey G Baranovskiy, Elena V Knatko, Fiona C Gray, Tahir H Tahirov, Stuart A MacNeill

**Affiliations:** 1 Wellcome Trust Centre for Cell Biology, University of Edinburgh, King's Buildings, Mayfield Road, Edinburgh, EH9 3JR, UK; 2 Department of Biology, University of Copenhagen, Københavns Biocenter, Ole Maaløes Vej 5, 2200 København N, Denmark; 3 Eppley Institute for Research in Cancer and Allied Diseases, University of Nebraska Medical Center, Omaha, Nebraska, USA

## Abstract

**Background:**

DNA polymerase δ plays an essential role in chromosomal DNA replication in eukaryotic cells, being responsible for synthesising the bulk of the lagging strand. In fission yeast, Pol δ is a heterotetrameric enzyme comprising four evolutionarily well-conserved proteins: the catalytic subunit Pol3 and three smaller subunits Cdc1, Cdc27 and Cdm1. Pol3 binds directly to the B-subunit, Cdc1, which in turn binds the C-subunit, Cdc27. Human Pol δ comprises the same four subunits, and the crystal structure was recently reported of a complex of human p50 and the N-terminal domain of p66, the human orthologues of Cdc1 and Cdc27, respectively.

**Results:**

To gain insights into the structure and function of Cdc1, random and directed mutagenesis techniques were used to create a collection of thirty alleles encoding mutant Cdc1 proteins. Each allele was tested for function in fission yeast and for binding of the altered protein to Pol3 and Cdc27 using the two-hybrid system. Additionally, the locations of the amino acid changes in each protein were mapped onto the three-dimensional structure of human p50. The results obtained from these studies identify amino acid residues and regions within the Cdc1 protein that are essential for interaction with Pol3 and Cdc27 and for *in vivo *function. Mutations specifically defective in Pol3-Cdc1 interactions allow the identification of a possible Pol3 binding surface on Cdc1.

**Conclusion:**

In the absence of a three-dimensional structure of the entire Pol δ complex, the results of this study highlight regions in Cdc1 that are vital for protein function *in vivo *and provide valuable clues to possible protein-protein interaction surfaces on the Cdc1 protein that will be important targets for further study.

## Background

Three evolutionarily-conserved DNA polymerase enzymes play essential roles at the eukaryotic replication fork [1]. DNA polymerase α-primase is essential for the initiation of leading strand synthesis at individual replication origins and for the initiation of each Okazaki fragment on the discontinuously-synthesised lagging strand. Extending from the RNA-DNA primers laid down by DNA polymerase α (Pol α)-primase, recent results indicate that DNA polymerase ε (Pol ε) is likely to replicate the bulk of the leading strand [2] while DNA polymerase δ (Pol δ) synthesises the bulk of the lagging strand [3], reviewed by [4,5]. In the absence of catalytically-active Pol ε, Pol δ is apparently able to synthesise both leading and lagging strands [6]. Completion of lagging strand synthesis requires subsequent processing of the nascent Okazaki fragments to remove the RNA primer and ligation to form a continuous DNA strand [1].

Each of the three replicative polymerases α, δ and ε is a member of the B family of DNA polymerases [1] and each is a multi-subunit entity, comprising an essential catalytic subunit and a number of smaller subunits, some of which are also essential for cell viability based on the results of genetic analysis in the yeasts [1]. Until recently, very little high-resolution three-dimensional structural information on the three polymerases was available, as the only solved structures were those of a 38 amino acid zinc finger derived from the carboxy-terminal end of the catalytic subunit of human Pol α and 75 amino acids of the amino-terminal domain of the B-subunit of human Pol ε, both determined by NMR [7,8]. A low-resolution structure of the budding yeast Pol ε complex was also obtained by cryo-EM reconstruction [9]. However, high-resolution structures are now available for the complex of p50 (the second subunit) and the N-terminal 144 amino acids of p66 (the third subunit) of human Pol δ (designated p50•p66_N_) [10,11] and for the complex of the C-terminal domain of budding yeast Pol1 protein, the catalytic subunit of Pol α, and the C-terminal PDE and OB fold domains of the Pol α B-subunit Pol12 [12].

The fission yeast *Schizosacchromyces pombe *is an excellent model for studying Pol δ structure and function [1]. In this organism, DNA polymerase δ is a heterotetrameric complex, comprising the catalytic subunit Pol3 and three smaller subunits Cdc1, Cdc27 and Cdm1 [13,14]. Pol3, Cdc1 and Cdc27 are essential proteins [15-18] whereas Cdm1 is not [19]. Orthologues of all four subunits make up human Pol δ. In contrast, Pol δ in the budding yeast *Saccharomyces cerevisiae *is a three-subunit enzyme; no Cdm1 orthologue is present in this organism. Pol3 binds directly to the B-subunit Cdc1 [20] via one of two conserved zinc finger modules located at the C-terminus of Pol3 [21]. Cdc1 binds the C-subunit Cdc27 [20] which interacts directly with two other key components of the replisome – the catalytic subunit of Pol α-primase [22] and the sliding clamp processivity factor PCNA [23]. Cdc27 has an unusual structure: a globular N-terminal domain of ~160 amino acids is followed by a highly extended C-terminal region of ~210 amino acids [14]. The interactions with Pol α-primase and PCNA both involve short conserved protein-protein interaction motifs located within this C-terminal region, but neither interaction is essential for replisome function [22,24]

**Figure 1:**
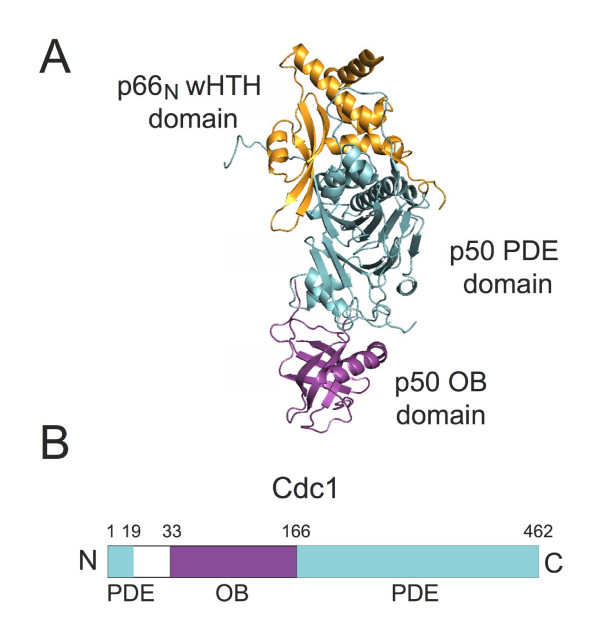
**Structure of the human p50•p66_N _complex**. A. Structure of the human p50•p66N complex showing the p50 PDE and OB domains (cyan and magenta respectively) and the p66N wHTH domain (gold). The figure was prepared with PyMol software (Delano Scientific) using the coordinates of p50•p66_N _complex (Protein Data Bank with accession code 3E0J) [10]. B. Schematic showing the predicted location of the PDE and OB domains in fission yeast Cdc1 in relation to their sequence numbering.

In contrast to PCNA and Pol α-primase, Cdc1 binds the N-terminal domain of Cdc27 [23]. The structure of p50•p66_N _complex [10,11] (where p50 and p66 are human orthologues of Cdc1 and Cdc27) now enables the mapping of the interactions within the Cdc1Cdc27 assembly. Figure 1 provides a summary view of the human p50•p66_N _complex (Figure 1A) together with a schematic representation of the domain structure of the two proteins in humans and yeast (Figure 1B). Three domains are apparent: p50 comprises a phosphodiesterase-like domain (PDE domain) and an oligonucleotide/oligosaccharide binding domain (OB domain) while p66_N _comprises a winged helix-turn-helix (wHTH) domain. The PDE domain is located in the centre of the complex with the OB domain on one side and the wHTH on the other (Figure 1A). The PDE and OB domains are present in the B-subunits of all three eukaryotic replicative polymerases as well as in the essential second subunit of the archaeal DNA polymerase PolD [25,26] found exclusively in the Euryarchaeota and Thaumarchaeota phyla [27]. In the case of the euryarchaeal proteins the PDE domain is active as a 3'-5' exonuclease, affording the PolD enzyme proofreading activity. Key residues required for nuclease activity are absent from the eukaryotic proteins; in the case of Pol δ and Pol ε, proofreading activity resides in the catalytic subunit of the complex [1].

This study describes the results of mutational analysis of fission yeast Cdc1. Despite its essential cellular role and widespread evolutionary conservation, the precise biochemical function of the Cdc1/p50 protein is not known nor is it clear how the structure of the protein is related to this. In fission yeast, a small number of temperature-sensitive mutant alleles of *cdc1 *have been isolated and characterised, and several truncated Cdc1 proteins tested for function *in vivo *[20]. The temperature-sensitive mutations were shown to result from single nucleotide changes in the gene sequence, resulting in a single amino acid changes in the encoded proteins. These changes mapped towards the C-terminus of the Cdc1 protein, within the PDE domain [20]. Similar results were seen with budding yeast *pol32*/*hys2 *mutations [28,29]. Analysis of truncated mutant Cdc1 proteins showed that removal of 20 amino acids from the C-terminus of Cdc1 abolished the *in vivo *function of the protein, while removal of the first 25 amino acids from the N-terminus led to dominant negative cell cycle arrest that could be reversed by simultaneous overproduction of Cdc27, suggesting that the mutant Cdc1-NΔ2–25 protein exerted its function by titrating Cdc27 from the cell [20].

To investigate further the structure and function of Cdc1, a collection of thirty mutant *cdc1 *alleles were generated by directed and random mutagenesis methods. Each of the mutations was mapped onto the three-dimensional structure of human p50 [10] and each mutant protein was then tested for function in fission yeast cells, and for binding to Pol3 and Cdc27 using the two-hybrid system.

## Results

### Pentapeptide insertion mutagenesis

Random mutagenesis of the *cdc1*^+ ^gene was carried out using the pentapeptide scanning mutagenesis (PSM) method [30,31]. This method makes use of a modified form of the Tn*3*-related transposon Tn*4430 *of *Bacillus thuringiensis*. Tn*4430 *transposes efficiently in *E.coli *and in doing so duplicates 5 bp of host sequence at the insertion point. The transposon also contains *Kpn*I restriction sites 5 bp from the outer end of its terminal repeats. If, following insertion of Tn*4430 *into the target gene, the bulk of the transposon is deleted by *in vitro Kpn*I restriction and re-ligation, a 15 bp sequence insertion remains. Since Tn*4430 *inserts with low sequence specificity, the pentapeptide insertions will vary in composition. The usefulness of this system has been demonstrated through its application for random mutagenesis of, amongst others, TEM-1 β-lactamase [30,32], XerD recombinase [33] and phospholipase D1 [34].

**Figure 2:**
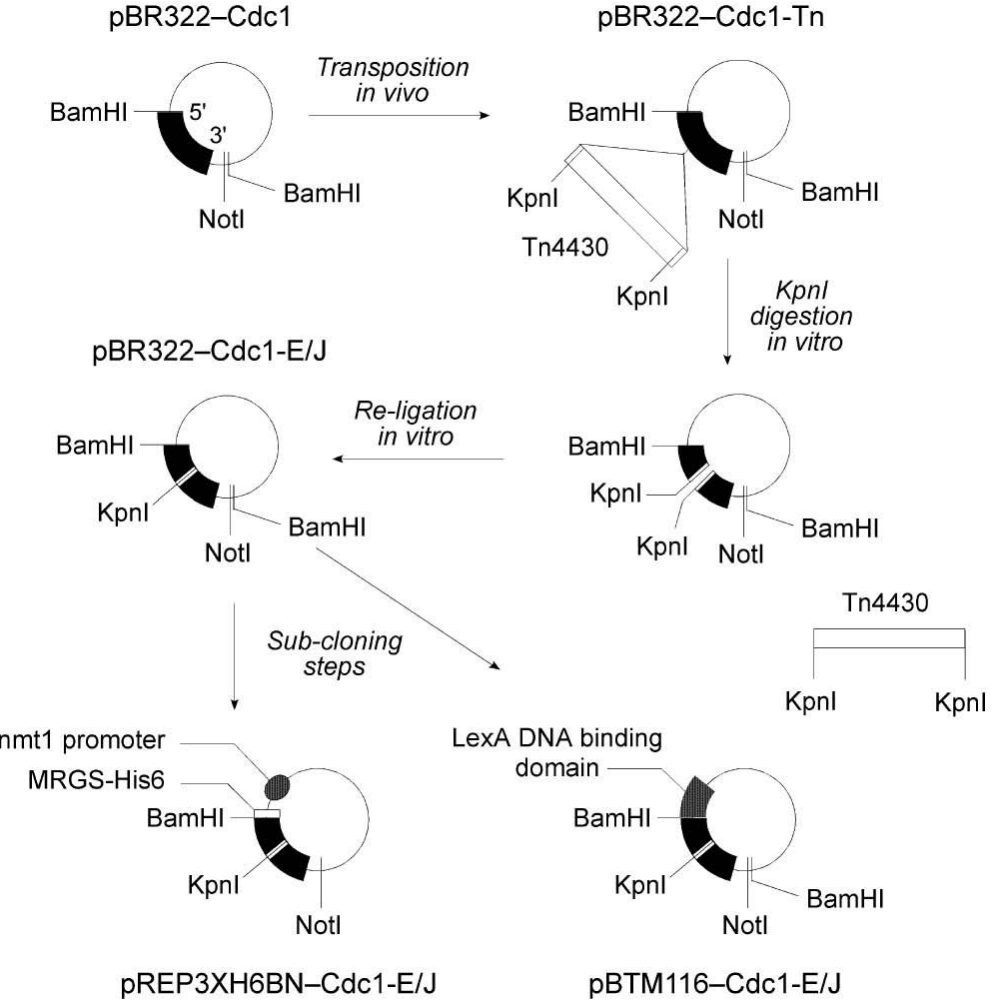
**Pentapeptide scanning mutagenesis (PSM) strategy**. A schematic representation of the mutagenesis and cloning strategies used to create the *cdc1 *PSM alleles. Plasmid pBR322-Cdc1 (top left) carries a *cdc1*^+ ^cDNA on a BamHI-BamHI fragment. This plasmid was transformed into *E.coli *FH1046 containing pHT385. Following mating with *E.coli *DS941(see Methods), plasmids in which the Tn4430 transposon had inserted into the *cdc1*^+ ^ORF (indicated as pBR322-Cdc1-Tn, top right) were identified by restriction mapping and the bulk of the Tn4430 sequences excised by KpnI digestion and self-ligation of the digested plasmids. The mutant alleles were then transferred into pREP3XH6BN, a fission yeast expression vector, and either pBTM116 or pBTM116BN, budding yeast two-hybrid bait vectors.

The strategy for mutagenesis of *cdc1*^+ ^is outlined in Figure 2. Plasmid pBR322-Cdc1 (see Methods) was transformed into *E.coli *FH1046 containing plasmid pHT385 which carries the Tn*4430 *transposon [30]. Individual transformant colonies were then mated with *E.coli *DS941 and resistant colonies identified and plasmids recovered. Restriction mapping was then used in order to identify clones in Tn4430 had inserted into *cdc1*^+ ^rather than the plasmid backbone, and the bulk of the transposon sequences then removed by cleavage with *Kpn*I followed by ligation under conditions that promoted self-ligation (see Methods).

### Number and distribution of insertion mutations

**Figure 3:**
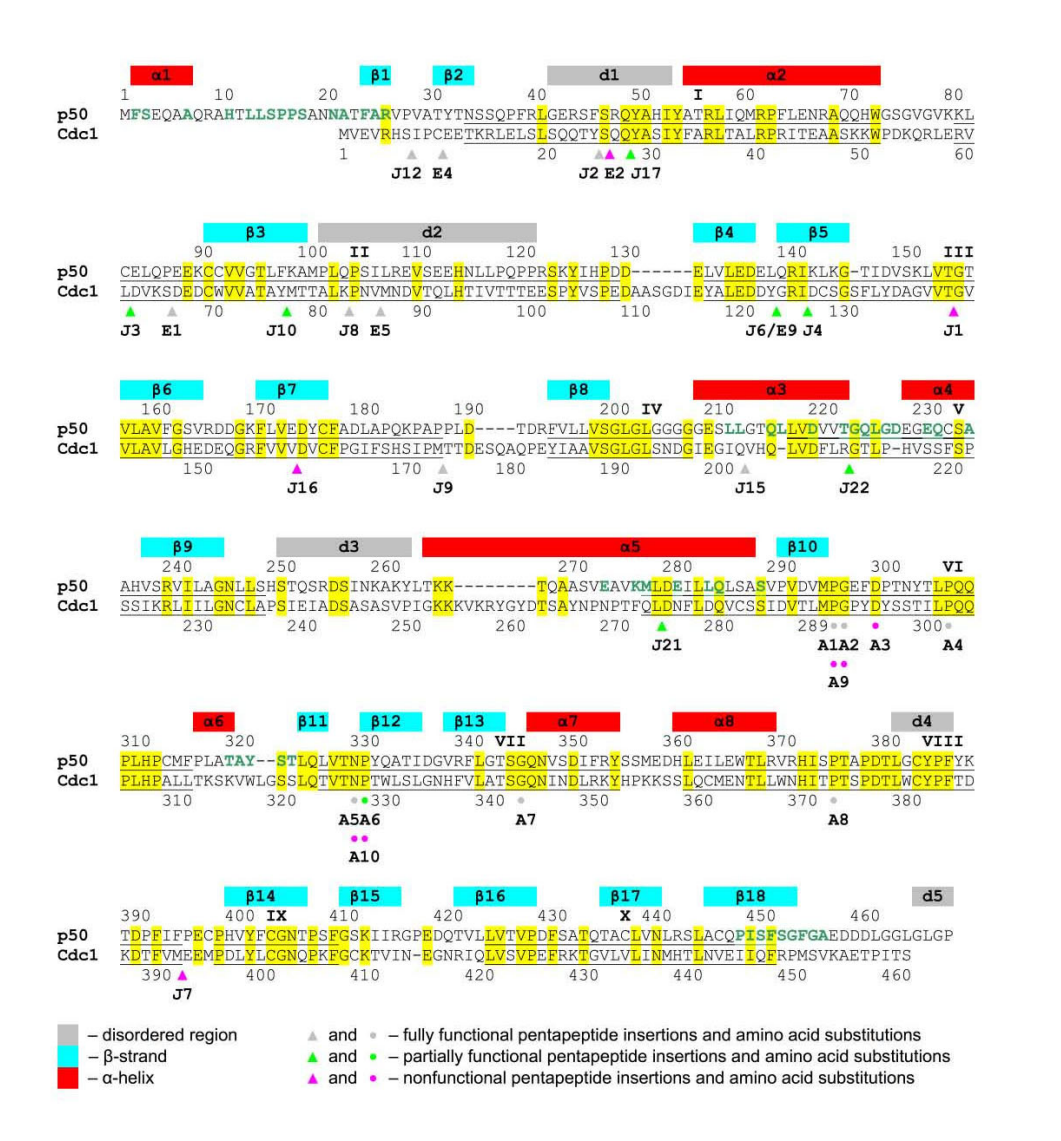
**Location of mutants in Cdc1 protein**. Sequence alignment of the fission yeast Cdc1 protein and its human orthologue p50 in single-letter code. Residues identical between the two proteins are highlighted in yellow. The sites of insertions and point mutations in Cdc1 are indicated by colour-coded triangles (pentapeptide insertions, E and J mutants) and circles (point mutations, A mutants). Grey circles/triangles indicate fully-functional mutants; green circles/triangles indicate partially-functional mutants; magenta circles/triangles indicate non-functional mutants. Note that the A9 and A10 mutants see two adjacent amino acids mutated and are therefore indicated by side-by-side (magenta) circles. Where the same insertion was found more than once, only one allele appears on the figure (see Table 1 for further information). The ten conserved regions defined in the previous study [36] are underlined and indicated by Roman numerals (I – X). The red, cyan and grey bars correspond to positions of α-helices, β-strands and disordered regions in human p50 [10]. The amino acid residues in the p50 protein that are involved in interactions with p66_N _are shown in green type. See text for details.

In total, 31 mutant alleles were generated by the PSM method (Table 1, Figure 3). Despite their independent origins, DNA sequence analysis revealed that these corresponded to only 20 different mutant alleles (Table 1), indicating that insertion of Tn*4430 *is not entirely random, as noted previously [30]. The insertions also clustered towards the 5' end of the *cdc1*^+ ^gene, with 26 of the 31 insertions mapping within the first 420 bp of the 1386 bp ORF. Clustering towards the 5' end of the ORF was observed in a similar study of the *S.pombe rfc2*^+ ^gene cloned into pBR322 [35]. Thirty of the mutant alleles contained a 15 nt insertion when compared with the wild-type *cdc1*^+ ^sequence. The remaining allele (Cdc1-E2) contained a 16 nt insertion, presumably the result of an inexact transposition event. This insertion leads to the Cdc1 protein being truncated after amino acid 26. Another insertion also led to production of a truncated protein: Cdc1-J17 was truncated after amino acid 27. Two of the insertions resulted in different insertions at the same amino acid position in the protein (Cdc1-J6 and Cdc1-E9, between amino acids 124 and 125).

**Table 1:** Pentapeptide insertion and site-directed mutants: a summary

**Mutant**	**Sub-domain**	**Location**	**Inserted sequence or point mutation(s)**	**Ability to substitute for Cdc1 at different expression levels**		**Interaction with Pol3 and Cdc27 by two-hybrid analysis (%)**	
				**Low**	**High**	**Pol3**	**Cdc27**

WT				+	+	100	100

J12	N	7	Ser-GlyValProHisSer-Ile	+	+	<1	59

E4	N	10	Cys-GlyValProProCys-Glu	+	+	16	62

J2	I	25	Tyr-ArgGlyThrProTyr-Ser	+	+	46	<1

E2	I	26	Ser-GlyValProLysTer	-	-	<1	<1

J17	I	28	Gln-Ter	-	+	2	<1

J3	I/II	62	Leu-GlyValProLeuLeu-Asp	-	+	54	<1

E1	I/II	66	Ser-GlyValProGlnSer-Asp	+	+	65	1

J10	II	77	Tyr-ArgGlyThrProTyr-Met	-	+	<1	30

J8	II	83	Lys-GlyValProLeuLys-Pro	+	+	<1	128

E5	II	86	Val-MetGlyTyrProVal-Met	+	+	48	88

J6	III	124	Tyr-GlyValProHisTyr-Gly	-	+	<1	<1

E9	III	124	Tyr-GlyGlyTyrProTyr-Gly	-	+	24	<1

J4	III	127	Ile-GlyValProArgIle-Asp	-	+	36	<1

J1	III	141	Thr-GlyValProLeuThr-Gly	-	-	<1	<1

J16	III/IV	160	Val-GlyGlyThrProVal-Asp	-	-	<1	<1

J9	III/IV	174	Met-ArgGlyThrProMet-Thr	+	+	91	102

J15	IV/V	203	Gln-GlyValProLeuGln-Val	+	+	1	78

J22	IV/V	212	Arg-GlyValProLeuArg-Gly	-	+	25	7

J21	V/VI	275	Leu-GlyValProGlnLeu-Asp	-	+	<1	<1

A1	VI	292	Pro292Ala	+	+	39	44

A2	VI	293	Gly293Ala	+	+	<1	19

A9	VI	292–293	Pro292Ala, Gly293Ala	-	-	<1	58

A3	VI	296	Asp296Ala	-	-	<1	23

A4	VI	303	Pro303Ala	+	+	<1	31

A5	VII	328	Asn328Ala	+	+	4	37

A6	VII	329	Pro329Ala	-	+	<1	6

A10	VII	328–329	Asn328Ala, Pro329Ala	-	-	<1	72

A7	VII	344	Gly344Ala	+	+	9	<1

A8	VIII	374	Pro374Ala	+	+	17	8

J7	VIII	393	Met-GlyValProLeuMet-Glu	-	-	<1	19

For the pentapeptide insertion mutants, multiply isolated insertions were as follows: J2 (re-isolated as J18, J20, E10), J3 (E3, E8), J10 (J11, E6, E7), J8 (J19) and J4 (J5, J13). The location of the insertions is given as the number of the last amino acid residue N-terminal to the insertion. The insertion sequences are shown between the hyphens. Note that the target site duplication results in the amino acid immediately N-terminal to the insertion being duplicated as the fifth amino acid of the insertion (except where stop codons are inserted) and that the insertions in Cdc1-J6 and Cdc1-E9 are found at the same amino acid but are of different sequence. Quantitative two-hybrid assay values are given as percentages of the interaction seen with wild-type Cdc1 with Pol3 (in the presence of Cdm1) or Cdc27. See Results for further details.

### Site-directed mutagenesis

In addition to the random pentapeptide insertion mutations, ten additional *cdc1 *mutations were constructed by oligonucleotide-directed *in vitro *mutagenesis (see Methods). Eight of these alleles resulting in the encoding proteins (Cdc1-A1 to -A8) differing from the wild-type Cdc1 by a single amino acid substitution. The remaining two alleles encoded proteins (Cdc1-A9 and Cdc1-A10) that featured substitutions of two adjacent amino acids. All the mutated amino acids were amongst those that are found conserved between the eukaryotic DNA polymerase B-subunits and the archaeal PolD small subunit [26,36]. Mutations A1 – A4 and A9, which combines mutations A1 and A2, cluster in conserved region VI [36], whereas mutations A5, A6 and A10, which combines A5 and A6, map to conserved region VII. A7 and A8 map to conserved regions VII and VIII, respectively (see Figure 3).

### Analysis of mutant function in fission yeast

#### Expression in *cdc1Δ* cells

In order to test for function in *S.pombe*, each allele was subcloned to plasmid pREP3XH6BN to permit expressing of the mutant proteins under the control of the thiamine-repressible *nmt1 *promoter (Figure 2, see Methods for details). The resulting plasmids were then transformed into a *cdc1*^+^/*cdc1::ura4*^+ ^h^-^/h^+ ^diploid strain and diploid transformants isolated at 32°C on supplemented EMM plates with and without 5 μg/ml thiamine. Transformant colonies were then picked and subjected to treatment with helicase to kill vegetative cells and degrade the walls of the asci present in the nutritionally-depleted colony centres. Following washing, the spores were plated on supplemented EMM media with and without 5 μg/ml thiamine and incubated at 32°C for 4–6 days (see Methods for further details).

Figure 3 summarises the properties of the Cdc1 mutants. When overexpressed (in cells grown in the absence of thiamine), twenty-three of the thirty mutant proteins were able to substitute for complete loss of wild-type Cdc1 function (grey/green circles/triangles in Figure 3). Given that all the pentapeptide insertion sequences include a proline residue that might be expected to have a significant effect on the secondary structure in the vicinity of the insertion, it is perhaps surprising that only seven of the insertions abolish Cdc1 function outright (purple circles/triangles in Figure 3). However, related studies of two other essential fission yeast DNA replication factors Rfc1 [37] and Rfc2 [35] have revealed similar ratios of functional to non-functional mutations, as have published studies of unrelated proteins [30,32-34]. Of the twenty-three functional alleles, nine were able to support growth only in cells grown in the absence of thiamine, indicating that high level expression of the mutant proteins is required for rescue (Figure 3, green circles/triangles).

#### Expression in *cdc1-P13* cells

In addition to testing the function of the mutant proteins in a *cdc1Δ *background, each was expressed in wild-type cells and in cells carrying the temperature-sensitive *cdc1-P13 *allele [15]. Expression of the mutant proteins had no detectable effect on wild-type cells. In *cdc1-P13 *cells, the proteins were tested for their ability to rescue at the restrictive temperature of 36.5°C in cells grown in the presence and absence of thiamine. The results obtained broadly mirrored those seen with the *cdc1Δ *strain but with the following exceptions: at low expression levels, several of the mutant proteins that could not rescue *cdc1Δ *could rescue *cdc1-P13 *at 36.5°C, namely Cdc1-A6, -J3, -J4, -J5, -J10, -J11, -J13 and -J21, and two of the mutants, Cdc1-A10 and -J7, displayed dominant negative properties at 28°C, described further below.

### Analysis of protein-protein interactions using the two-hybrid system

**Figure 4:**
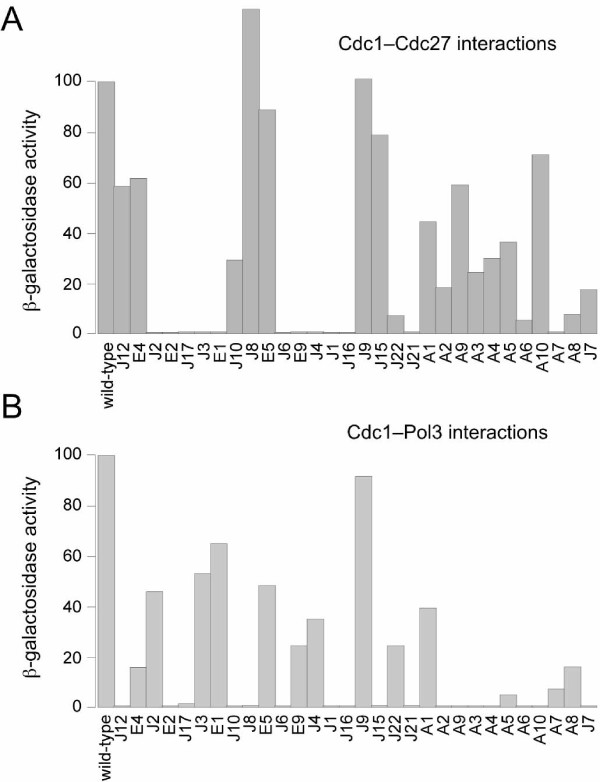
**Quantitative two-hybrid analysis**. A. The Cdc1 mutants, arranged depending on the position of the mutation within the Cdc1 protein, from N- to C-terminus, were tested for their ability to interact with Cdc27 using the two-hybrid system in *S.cerevisiae *CTY10-5d. Quantitative values were obtained using an ONPG-based β-galactosidase assay from liquid cultures (see Methods). Values are expressed in arbitrary units with the wild-type Cdc1-Cdc27 interaction being set at 100 units (~65 Miller units of β-galactosidase activity, see [20]). B. Similar analysis of Cdc1-Pol3 interactions, performed in the presence of Cdc27 (see text).

As described in the Introduction, the Cdc1 protein interacts directly with both the catalytic subunit of the Pol δ complex Pol3 and also with the C-subunit Cdc27. These interactions can be conveniently monitored using the yeast two-hybrid system [20,23]. Each *cdc1 *mutant allele was therefore cloned into the two-hybrid bait pBTM116 (or the related pBTM116BN plasmid) to allow expression of Cdc1 fused to the DNA binding domain of bacterial LexA protein (see Methods). The resulting plasmids were initially co-transformed into *S.cerevisiae *CTY10-5d along with the prey plasmid pGAD2F-Cdc27, which expresses Cdc27 fused to the yeast Gal4 transcriptional activation domain. Co-transformants were monitored for β-galactosidase activity by X-gal overlay assay (data not shown) and ONPG liquid assay (Figure 4A).

To examine whether the mutant Cdc1 proteins interacted with Pol3, each pBTM116-Cdc1 plasmid was transformed into CTY10-5d along with the prey plasmid pACT-Pol3 and pAA-Cdc27. The latter directs expression of Cdc27 as a c-myc epitope-tagged fusion protein. Previously it was shown that interaction between LexA-Cdc1 and Gal4-Pol3 could only be detected when Cdc27 was simultaneously co-expressed in the cells [23]. Presumably Cdc27 binds to Cdc1 to stabilise the protein or alter its conformation, allowing interaction with Pol3 to take place at a level that can be detected under two-hybrid assay conditions. As above, β-galactosidase activity was monitored by X-gal overlay assay (data not shown) and ONPG liquid assay (Figure 4B). Those mutant Cdc1 proteins that were unable to bind to Cdc27 in the two-hybrid system would be predicted to be also incapable of binding Pol3.

### Analysis of dominant negative Cdc1 mutants

**Figure 5:**
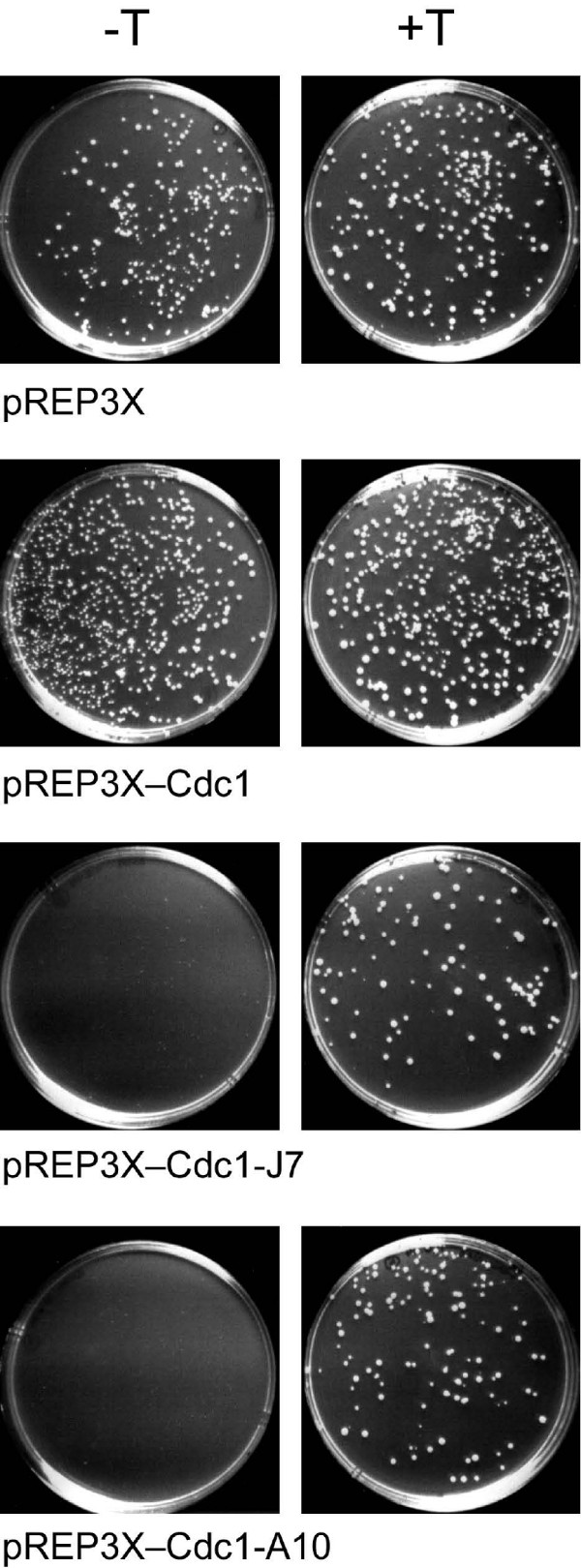
**Dominant negative Cdc1 mutants**. *cdc1-P13 *cells carrying plasmids pREP3X, pREP3XH6BN-Cdc1, pREP3XH6BN-Cdc1-J7 or pREP3XH6BN-Cdc1-A10 were grown up in EMM medium at 28°C before being plated onto EMM plates with (+T, right hand panels) or without 5 μg/ml thiamine (-T, left hand panels) and incubated for 4 days at 28°C.

Two of the Cdc1 mutant proteins exerted a strong dominant negative phenotype when expressed at high level in *cdc1-P13 *cells. Transformed *cdc1-P13 *cells carrying either pREP3XBN-Cdc1-J7 or -A10 were viable at 28°C only when grown on medium containing thiamine to repress the nmt1 promoter (Figure 5). When transferred to medium lacking thiamine, high-level expression of the mutant proteins led to cell cycle arrest (elongated cell phenotype) and a failure to form colonies. Similar behaviour was observed when these mutant proteins were expressed in *cdc1–223 *cells at 28°C, although the phenotype was somewhat weaker (data not shown).

The two-hybrid analysis described above (Figure 4) suggests that both Cdc1-J7 and Cdc1-A10 retain the ability to bind to Cdc27 but cannot bind Pol3, suggesting that the dominant negative phenotype may be the result of the mutant proteins interacting with Cdc27 to form non-functional Cdc1-Cdc27 dimers, thus titrating Cdc27 from the cell, impairing Pol3-Cdc1-Cdc27 (Pol δ) complex formation and inhibiting chromosome replication. If this were the case, simultaneous overproduction of Cdc27 would be expected to reverse the toxic effects of Cdc1-J7 and Cdc1-A10, by providing excess Cdc27 to interact with non-mutant Cdc1.

To test this, *cdc1-P13 *cells expressing Cdc1-J7 and Cdc1-A10 were co-transformed with plasmid pREP4X-Cdc27 which expresses the full-length Cdc27 protein, also from the nmt1 promoter [23]. However, no rescue of the dominant negative phenotype was observed: *cdc1-P13 *cells expressing Cdc27 and either Cdc1-J7 and Cdc1-A10 were still unable to grow on plates lacking thiamine at 28°C. We also tested whether overproduction of Pol3 or a nuclear-targeted form of the C-terminal ZnF domain of Pol3 would rescue the toxic effects of Cdc1-J7 and Cdc1-A10, despite their apparent inability to bind Pol3 as gauged by two-hybrid assay (Figure 4): again, no rescue was seen. Previously, overproduction of an N-terminally truncated form of Cdc1, Cdc1-NΔ25, has been shown to exert a similar effect on *cdc1-P13 *cells [20]. In the case of Cdc1-NΔ2–25, however, simultaneous overproduction of Cdc27 was sufficient to rescue the phenotype. This suggests that the Cdc1-J7 and -A10 proteins exert their effects through a mechanism distinct from that of Cdc1-NΔ2–25, perhaps by binding to, and titrating out, an as yet unidentified Pol δ interacting factor. Screening for high copy suppressors of the Cdc1-J7 and Cdc1-A10 dominant negative phenotypes might allow this factor to be identified, although negative regulators of the nmt1 promoter are also likely to be identified in such a screen.

## Discussion

In this report, we describe the results of mutational analysis of the fission yeast Cdc1 protein using both random and site-directed mutagenesis techniques. In total, thirty mutant proteins were expressed and assayed for function *in vivo *and for binding to Pol3 and Cdc27 by two-hybrid. The results of these assays are summarised in Table 1.

**Figure 6:**
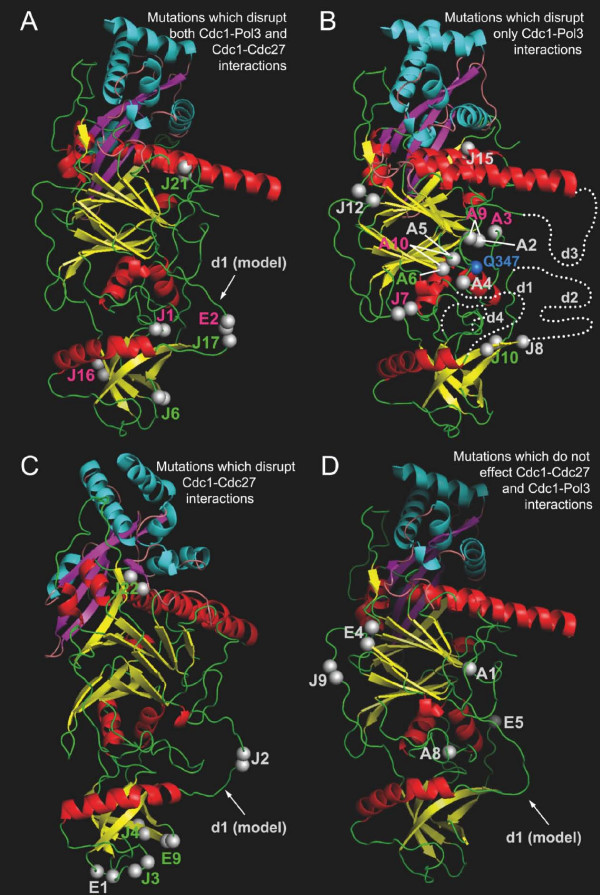
**Mapping of Cdc1 mutants to the structure of human p50•p66_N _complex**. The secondary structure elements are colour-coded as follows: α-helices, β-strands and coils are red, yellow and green in p50, and cyan, magenta and light pink in p66_N_. The sites of pentapeptide insertions and amino acid substitutions are highlighted by double and single grey spheres, respectively. In panel B the blue sphere indicates the position of Gln347. The labels in magenta, green and grey correspond to non-functional, partially functional and fully functional pentapeptide insertions or amino acid substitutions. The disordered region d1 was modelled for panels A, C, and D. The positions of disordered regions d1-d4 are drawn by dotted lines in panel B. The figure was prepared with PyMol software (Delano Scientific) using the coordinates of p50•p66_N _complex (Protein Data Bank with accession code 3E0J) [10].

For the purposes of the following discussion, we divide the mutations in four separate groups based on the two-hybrid interaction data presented in Figure 4 and use the structure of the human p50 protein [10] as the basis for discussion of possible structural effects of the mutations. In the absence of a crystal structure for the fission yeast Cdc1 protein, the human p50 structure represents the only available option for this type of analysis but it is important to note that the two proteins are only ~35% identical at the primary sequence level and that this could affect some of the conclusions drawn. An additional point to note, particularly when considering mutations that appear to disrupt Cdc1 function altogether, is that possible effects of individual mutations on protein stability have not been addressed in this study. For technical reasons we have been unable determine the expression levels of the mutants, even in cells cultured in the absence of thiamine. Figure 6 shows the locations of the mutations in each group mapped onto the structure of human p50 according to the protein sequence alignment shown in Figure 3.

### Mutations that disrupt both Pol3-Cdc1 and Cdc1-Cdc27 interactions

The E2, J1, J16, J21, J17 and J6 mutations disrupt Cdc1 interactions with both Pol3 and Cdc27 in two hybrid-assays (Figure 4); the locations of the mutated residues on the three-dimensional structure of human p50 are shown in Figure 6A.

Five of the six mutations in this group are pentapeptide insertions (J1, J16, J21, J17 and J6, see Table 1). The J21 insertion is in the middle of α5 (Figure 6A) and likely interferes with the folding of this helix, which is an important feature of PDE domain. Several residues within α5 are also involved in Cdc27 binding, raising the possibility that loss of Pol3-Cdc1 interaction in two-hybrid assays is the caused by disruption of the Cdc1-Cdc27 interaction since Pol3-Cdc1 interactions were only detectable by two-hybrid in yeast cells co-expressing Cdc27. In contrast, the J1, J6 and J16 insertions are located within, and appear likely to disrupt the folding of, the OB domain (Figure 6A). The fact that none of the three latter insertions are close to the Cdc1-Cdc27 interface (based on the p50•p66_N _structure) appears to rule out the possibility that Pol3-Cdc1 two-hybrid interaction is impaired by direct modification of Cdc1-Cdc27 interaction interface. Instead, the disruption of OB domain fold by J1, J6 and J16 insertions is likely to affect the Cdc1-Cdc27 interaction in part by destabilizing the PDE domain and in part by preventing the correct positioning of N-terminal residues which are important for the function of Cdc1 [20]. The consequences of the J1 and J16 mutations for Cdc1 activity *in vivo *were more severe than for J6 and J21, since neither J1 nor J16 was able to substitute for loss of Cdc1 in *cdc1Δ *cells under any conditions, while J6 and J21 were able to rescue when overexpressed (Figure 3).

For the remaining two mutations in this group (J17 and E2), it is not surprising these the mutant protein fail to interact with both Pol3 and Cdc27, as both mutations result in incorporation of a stop codon into the *cdc1 *sequence and premature termination of translation in the flexible β_2_α_2 _linker, after amino acids 26 and 28 respectively. When expressed at near wild-type levels in *S.pombe*, from the repressed *nmt1 *promoter, neither protein is able to support growth of *cdc1Δ *cells. However, when overexpressed (*nmt1 *promoter depressed in the absence of thiamine), *cdc1Δ *cells expressing Cdc1-J17 were able to grow (Figure 3). This is most likely a consequence of low-level readthrough of the amber (UAG) stop codon in the *cdc1-J17 *allele. Although E2 is also an amber mutation, the sequence insertion in this allele comprised 16 nucleotides rather than the usual 15, so that expression of a full-length Cdc1 protein would require the unlikely combination of readthrough of the AUG codon *and *translational frameshifting.

### Mutations that disrupt Pol3-Cdc1 interactions only

This group contains a total of thirteen mutations that can be further divided into two sub-groups on the basis of the ability to substitute for wild-type Cdc1 in *cdc1Δ *cells. The first sub-group contains six mutations that display either impaired function *in vivo *(rescuing *cdc1Δ *cells only when overexpressed from the derepressed *nmt1 *promoter) or which cannot rescue *cdc1Δ *cells under either of the conditions tested. This sub-group comprises the pentapeptide insertions J7 and J10, the single point mutations A3 and A6, and the double point mutations A9 and A10 (Figure 6B). A9 is a combination of A1 and A2; A10 of A5 and A6 (Figure 3). Five of the six mutations are located in the PDE domain and one (J10) in the OB domain. Each of the six mutant proteins is able to bind Cdc27 in two-hybrid (although in the case of A6 binding is reduced to <10% of that seen with the wild-type Cdc1 protein) but is greatly impaired in Pol3 binding ability (< 0.5% of binding seen with wild-type protein, see Figure 4). Strikingly, all six mutations map to the same side of Cdc1, identifying a possible Pol3-interacting surface. In support of this, glutamine 345 in human p50 (glutamine 347 in Cdc1) also maps to this region (indicated in Figure 6B). A mutation in the corresponding residue in budding yeast Pol31 (lysine 358 mutated to glutamate) was identified in a screen for dominant extragenic suppressors of the temperature-sensitive *pol3–13 *mutation, in which one of four cysteines of the second C-terminal zinc finger of Pol3 (designated ZnF2) is replaced by serine [38,39]. Subsequent work showed that the ZnF2 domain was both necessary and sufficient for Pol31 binding by Pol3 and that in two-hybrid assays, the K358E mutant of Pol31 interacted more strongly with both wild-type and mutant forms of ZnF2 [21]. Taken together, these results suggest that the region defined by these mutations constitutes a Pol3 binding site on Cdc1. This suggestion is consistent with the recent crystal structure of interacting domains of the budding yeast Pol1 and Pol12 proteins, the catalytic and B-subunits of Pol α respectively [12]. The C-terminal domain of Pol1, comprising the two zinc fingers ZnF1 and ZnF2 and a three α-helix bundle, makes extensive contacts with the PDE and OB fold domains of Pol12 over a strikingly large area that is equivalent to that defined by our study of Cdc1-Pol3 interactions (Figure 6B). Intriguingly, the disordered regions d1-d4 in the human p50 structure (indicated by the grey bars in Figure 3) are localised to the same region [10]. It is tempting to speculate that these disordered regions also have a part to play in binding Pol3 and become structured only once Pol3 is bound.

The second sub-group of mutations unable to bind to Pol3 comprises mutations J8, J12, J15, A2, A4 and A5. These display >20% binding to Cdc27 in the Cdc1-Cdc27 two hybrid assay but with the exception of A5, display < 0.5% binding to Pol3 in the Pol3-Cdc1-Cdc27 two hybrid assay (A5 displayed ~5% binding compared to wild-type Cdc1, Figure 4). Despite their extremely limited ability to bind to Pol3, however, these mutant proteins were able to function *in vivo*, even when expressed at near wild-type levels from the repressed nmt1 promoter (Figure 3). The reason for this discordance is unclear but one possibility is that the mutants benefit from the presence of the fourth subunit of the Pol δ complex in fission yeast, Cdm1 [19]. In both fission yeast and in mammalian cells, the Cdm1 protein (designated p12 in mammals) appears to have a stabilising effect on the Pol δ complex [13,40-42] possibly through direct binding to both Pol3 and Cdc1 [43]. There is no Cdm1 orthologue in the budding yeast *S.cerevisiae*, the host for the two-hybrid analysis presented here [44]. Mapping of the J8, J12, J15, A2, A4 and A5 mutations onto the structure of human p50 reveals that J8, A2, A4, and A5 may directly affect the disordered loops (Figure 6B), which we propose to form an area of protein-protein interactions. The remaining two mutations, J12 and J15, are apart from the suspected interaction area and are likely to exert their influence to Pol3-Cdc1 interactions via an allosteric effect: J12 via an N-terminal residues to a disordered region d1, and J15 via a loop β8-α3 to the disordered region d2.

### Mutations that disrupt Cdc27 interactions only

As noted above, in the two-hybrid assay system used here and described previously [21,23], Pol3-Cdc1 interactions were only detectable when Cdc27 was co-expressed in the reporter cells. Under these conditions, Cdc27 presumably serves to stabilise or alter the conformation of Cdc1 to facilitate its binding to Pol3. It is therefore surprising that five of the insertion mutant Cdc1 proteins generated in this study (E1, J2, J3, E9, J4) were able to interact with Pol3 in the Pol3-Cdc1-Cdc27 two-hybrid assay (displaying >20% of wild-type binding activity) despite being unable to interact with Cdc27 in the Cdc1-Cdc27 assay. Interestingly, four of these map to the OB domain and the fifth (J2) to the β_2_α_2 _disordered linker. All five are therefore located some distance away from the Cdc1-Cdc27 interface (Figure 6C) making it unlikely that they have a direct effect on Cdc27 binding. It may be the case that disruption of the OB domain and β_2_α_2 _linker results in a widespread perturbation of the structure of Cdc1 and disruption of the Cdc1-Cdc27 interface. When Pol3 is present (as in the Pol3-Cdc1-Cdc27 two-hybrid assay), the structure of the Cdc1 protein, and more specifically the putative Pol3 binding region described in the preceding section, may be stabilised, effectively insulating the Cdc1-Cdc27 interface from the effects of disruption of the OB domain and β_2_α_2 _linker. Cdc1-Cdc27 interactions are also impaired by the J22 insertion (<10% binding in two-hybrid assay) which maps close to the Cdc1-Cdc27 interface (Figure 6C).

### Mutants unimpaired in Pol3 or Cdc27 binding

Five mutations fall into this group: the insertions E4, E5 and J9, and the single point mutations A1 and A8. Four of these (the exception is E4) map to loop regions in the human p50 protein (Figures 3 and 6D) and probably do not disrupt the secondary structure of Cdc1. The E4 insertion is on the N-terminus of β_2_. However, the two amino acids preceding the insertion site (proline 9 and cysteine 10) are identical with the residues at the positions 4 and 5 of the pentapeptide insertion (see Table 1). This has the effect of shifting the insertion from the N-terminal end of β_2 _to the β_1_β_2 _linker, which likely explains the low impact of this insertion.

## Conclusion

Using both site-directed and random mutagenesis strategies, thirty new mutant Cdc1 proteins have been test for *in vivo *function and for Pol3 and Cdc27 binding using the two-hybrid system. The results obtained can be rationalised in the context of the recently published crystal structure of human p50 bound to the N-terminal domain of human p66 [10] and, in addition, offer new insights into the potential location of interaction surfaces on Cdc1 for Pol3.

## Methods

### Bacterial strains and methods

*E.coli *FH1046 and DS941, used as the donor and recipient strains for pentapeptide mutagenesis, have been described elsewhere [30] and were the generous gift of Dr F. Hayes (now at UMIST, UK). Otherwise, *E.coli *JM109 and DH5α were used for routine cloning work. Standard molecular cloning methods [45] were used throughout, unless otherwise stated.

### Yeast strains and methods

The fission yeast strains used in this study were as follows: *cdc1–223 *(*mis1–223) his2 leu1–32 *h^+^, *cdc1–64 *(*mis1–64*) *ade6–704 leu1–32 *h^-^, *cdc1-A24 leu1–32 ura4-D18 *h^-^, *cdc1-P13 leu1–32 *h^+^, *cdc27-P11 leu1–32 *h^+^, *leu1–32 ura4-D18 *h^- ^and *cdc1*^+^/*cdc1::ura4*^+ ^*leu1–32/leu1–32 ura4-D18/ura4-D18 ade6-M210/ade6-M216 *h^-^/h^+ ^[20]. The *cdc1*/*mis1 *strains [46] were a generous gift of M.Yanagida (Kyoto University, Kyoto, Japan); *cdc1-A24 *was a generous gift of K.L. Gould (Vanderbilt Uinversity, Nashville, USA). Standard growth conditions [47] were used throughout. For two-hybrid analysis, *S.cerevisiae *CTY10-5d and standard methods were used [20].

### Pentapeptide mutagenesis method

For pentapeptide mutagenesis, a 1.5 kb BamHI fragment containing a *cdc1*^+ ^cDNA was sub-cloned from pBTM116-Cdc1 [20] into pBR322, at the unique BamHI site in the latter, to generate plasmid pBR322-Cdc1. pBR322 was chosen as it is a low copy number plasmid that is devoid of restriction sites for KpnI, two features that are important for the mutagenesis strategy [30]. The orientation of the *cdc1*^+ ^insert in pBR322-Cdc1 is such that the 3' end of the insert is proximal to the unique HindIII site in pBR322. Mutagenesis was carried out as described previously [30]. Briefly, pBR322-Cdc1 was transformed into the donor *E.coli *strain FH1046 containing the kanamycin-resistant Tn*4430*-carrying plasmid pHT385. Transformant (donor cell) colonies obtained after overnight growth at 37°C on LB agar plates containing kanamycin (50 μg/ml) and ampicillin (50 μg/ml) were picked and resuspended in 100 μl of LB broth (no antibiotics) and 2.5 μl spotted onto LB agar plates. Next, 2.5 μl aliquots of recipient cells (from an overnight culture of *E.coli *DS941 in LB supplemented with 50 μg/ml streptomycin) were applied to the donor spots, after which the plates were air-dried and incubated for 3 hours at 37°C to allow mating. The mating mixes were then resuspended in 300 μl of LB broth, and 100 μl plated onto LB agar plates containing ampicillin, kanamycin and streptomycin. Colonies were then screened either by preparing plasmid DNA and digesting this with BamHI to determine if the Tn*4430 *had inserted within the vector or the BamHI insert, or by PCR analysis of individual colonies using oligonucleotides flanking the BamHI site in pBR322, in which case those isolates in which a 1.6 kb band corresponding to a Tn*4430*-less *cdc1*^+ ^insert were discarded. Each pBR322-Cdc1-Tn plasmid was then digested with KpnI, re-ligated and transformed into *E.coli *JM109. Plasmids were prepared from ampicillin-resistant, kanamycin-sensitive colonies and checked for absence of the transposon by restriction mapping. The 1.5 kb BamHI-NotI fragments from the transposon-free plasmids were then cloned into plasmid pREP3XH6N (below) to facilitate expression in *S.pombe*, sequence analysis and subsequent sub-cloning to pBTM116.

### Oligonucleotide-directed mutagenesis

Oligonucleotide-directed *in vitro *mutagenesis was carried out using the Mutagene II *in vitro *mutagenesis system (BioRad). The template used was pTZ19R-Cdc1-cDNA [20]. Oligonucleotides used were as follows: mutant A1, oligonucleotide AANO1 (5'-TAGATGTCACTTTAATG**GCT**GGTCCTTATG-3', with mutant alanine codon underlined); A2, AANO2 (5'-GTCACTTTAATGCCT**GCT**CCTTATGATTAC-3'); A3, AANO3 (5'-CCTGGTCCTTAT**GCT**TACAGTTCAACTATC-3'); A4, AANO4 (5'-GTTCAACTATCCTT**GCT**CAACAGCCTTTGC-3'); A5, AANO5 (5'-ACAAACAGTTACG**GCT**CCCACTTGGCTTTC-3'); A6, AANO6 (5'-AAACAGTTACGAAT**GCC**ACTTGGCTTTCTC-3'); A7, AANO7 (5'-TGGCTACTAGC**GCC**CAAAACATTAATGATC-3'); A8, AANO8 (5'-AATCATATCACA**GCT**ACCAGCCCTGATACC-3'); A9, AANO1/2 (5'-GTCACTTTAATG**GCTGCT**CCTTATGATTAC-3'); A10, AANO5/6 (5'-ACAAACAGTTACG**GCTGCC**ACTTGGCTTTC-3'). The resulting mutants were then subcloned to pREP3XH6BN and either pBTM116 or pBTM116BN, as below.

### Expression in yeast

Each pentapeptide insertion allele was subcloned as a BamHI-NotI fragment from pBR322-Cdc1-Tn into pREP3XH6BN, a derivative of pREP3XH6 [48] in which a NotI site was introduced just 3' to the existing BamHI site by cutting the vector with BamHI and SmaI and ligating in a short linker made up from the following oligonucleotides: 3XH6Not-1 (5'-gatcCATCATC**GCGGCCGC**ATCG-3', with NotI site underlined and BamHI 5' overhang in lower case) and 3XH6Not-2 (5'-CGAT**CGCCGGCG**GATGATG-3'). When cloned into pREP3XH6BN, the Cdc1 protein is expressed as a hexahistidine fusion with the 13 amino acid sequence MRGSHHHHHHGIL N-terminal to the native initiating methionine [48]. The resulting plasmids were then transformed into *S.pombe cdc1*^+^/*cdc1::ura4*^+ ^*leu1–32/leu1–32 ura4-D18/ura4-D18 ade6-M210/ade6-M216 *h^-^/h^+ ^[20] by electroporation [49] and transformants obtained on EMM medium [47]. Individual colonies were then patched overnight at 32°C on ME medium to induce sporulation, before being treated overnight with SRP-Helicase enzyme (BioSepra, France) to break down the ascus walls and eliminate vegetative cells. Spores were then washed with water before being plated on EMM plates supplemented with adenine (EMM+A), uracil and adenine (EMM+AU), adenine and 5 μM thiamine (EMM+AT) and adenine, uracil and 5 μM thiamine (EMM+AUT) at 32°C. Leucine was omitted from all plates to facilitate selection of pREP3X plasmids which carry the *LEU2 *selectable marker. Adenine is required to permit the growth of haploid cells either the *ade6-M210 *or *ade6-M216 *alleles. The addition of uracil permits growth of *cdc1*^+ ^haploids; in the absence of uracil, only *cdc1::ura4*^+ ^haploids expressing functional Cdc1 proteins can grow. The presence of 5 μM thiamine represses the *nmt1 *promoter in pREP3X, reducing *cdc1 *expression by a factor of 80–100 compared to cells grown on EMM without thiamine. Following 4–6 days growth at 32°C, colonies were analysed by microscopy and by replica plating to confirm predicted genotypes.

### Two-hybrid analysis

For two-hybrid analysis, mutant *cdc1 *alleles were sub-cloned as a BamHI fragment into pBTM116 [20] or as a BamHI-NotI fragment to pBTM116BN, a modified form of pBTM116 in which a NotI site was added to the multiple cloning site by digesting pBTM116 with BamHI and PstI and ligating in a short duplex linker comprised of the following oligonucleotides: PBTMMOD1 (5'-gatcCGATCCG**GCGGCCGC**Ttgca-3', with NotI site underlined and BamHI and PstI overhangs, 5' and 3' respectively, in lower case) and PBTMMOD2 (5'-A**GCGGCCGC**CGGATCG-3'). The plasmids were then co-transformed into *S.cerevisiae *CTY10-5d along with either pGAD2F-Cdc27 (for analysis of Cdc1-Cdc27 interactions) or pGAD2F-Pol3 and pAA-Cdc27 (for analysis of Cdc1-Pol3 interactions). β-galactosidase activity was determined by an X-gal agar plate overlay assay and later by ONPG liquid culture assay, as previously described [20,21]. Liquid culture assays were done in triplicate with the values for individual cultures being within 10% of one another. The results shown in Figure 4 are mean values from the triplicate assays.

## Authors' contributions

The A and J mutant alleles were constructed and analysed in yeast by JSG who also performed all the two-hybrid analysis. The E mutant alleles were constructed and analysed in part by EVK. The remaining analysis was performed by FCG. AGB and THT contributed to the mapping of mutants to the three-dimensional structure of p50•p66_N _and analysis of the possible effects of Cdc1 mutations to Cdc1-Pol3 and Cdc1-Cdc27 interactions. The project was initiated and overseen by SAM. All authors read and approved the final manuscript.
